# Human dendritic cell activation induced by a permannosylated dendron containing an antigenic GM_3_-lactone mimetic

**DOI:** 10.3762/bjoc.10.133

**Published:** 2014-06-10

**Authors:** Renato Ribeiro-Viana, Elena Bonechi, Javier Rojo, Clara Ballerini, Giuseppina Comito, Barbara Richichi, Cristina Nativi

**Affiliations:** 1 Glycosystems Laboratory, Instituto de Investigaciones Químicas (IIQ), CSIC – Universidad de Sevilla, Américo Vespucio 49, 41092 Sevilla, Spain; 2 Department of NEUROFARBA, University of Florence, Viale Pieraccini 6, 50134 Firenze, Italy; 3 Department of Biochemical Science, University of Florence, Viale Morgagni 50, 50134 Florence, Italy; 4 Department of Chemistry, University of Florence, Via della Lastruccia 13, 50019 Sesto Fiorentino (FI), Italy

**Keywords:** cancer immunotherapy, DC-SIGN, DC targeting, glycodendron, GM_3_-lactone mimetic, multivalent glycosystems, multivalent interactions

## Abstract

Vaccination strategies based on dendritic cells (DCs) armed with specific tumor antigens have been widely exploited due the properties of these immune cells in coordinating an innate and adaptive response. Here, we describe the convergent synthesis of the bifunctional multivalent glycodendron **5**, which contains nine residues of mannose for DC targeting and one residue of an immunogenic mimetic of a carbohydrate melanoma associated antigen. The immunological assays demonstrated that the glycodendron **5** is able to induce human immature DC activation in terms of a phenotype expression of co-stimulatory molecules expression and MHCII. Furthermore, DCs activated by the glycodendron **5** stimulate T lymphocytes to proliferate in a mixed lymphocytes reaction (MLR).

## Introduction

Cancer immunotherapy [1] attempts to induce a long-lasting antitumor immunity and boost the immune response overcoming the tumor induced immunosuppression. The immune system, apart from very few exceptions, fails to taking an adequate course of action against tumors. Tumor cells are indeed poor antigen-presenting cells (APCs). Additionally, in neoplastic diseases the so-called “escape mechanisms” [2–4] enable tumor cells to elude tumor-bearing immunosurveillance of the host. A better understanding of the interactions between cancer and immune cells may lead to more efficient immunotherapy strategies [5–6].

In this context, the discovery of human cancer-specific antigens [7–8] has represented a challenge for the design of tailored cancer vaccines and it has allowed the development of antigen-specific immunotherapy strategies. This approach offers the advantage that the immune response induced by such antigens should presumably be limited to tumor cells bearing antigenic epitopes. To induce a persistent and efficient tumor immune response and generate a pool of tumor antigen specific activated immune cells, a complex cross-talk between the innate and the adaptive immune system is a prerequisite. In this context, during the last two decades, dendritic cells (DCs) have clearly been identified as essential candidates to generate therapeutic immunity against tumors [9–12].

DCs are the principal antigen-presenting cells (APCs) in the immune system where they play a central role because they are able to control self-tolerance as well as induce an effective immune response [13–14]. They provide an essential link between innate and adaptive immune responses [13]. They survey the environment and, based on the typical non-clonal recognition receptors of the innate immune system, they take up the non-self agents and transmit the resulting information to both B and T cells of the adaptive immune system. DCs contribute to the peripheral tolerance and this might be determined by their functional status. Therefore, DC activation is crucial to their function. During activation, DCs up-regulate MHCII molecules and co-stimulatory factors, both of which are mandatory to achieve a complete immunostimulatory function. Since the discovery of their key role in immunogenicity in 1973 by R. Steinman [15], DCs have been identified as “nature’s adjuvants”. Today, they are considered natural targets for antigen delivery and therapeutic vaccination against cancer [9–10].

Several approaches have been investigated to pulse DCs with target antigens with the aim to induce robust and long-lasting CD4+ and CD8+ T cell responses against tumors [9–10]. In general, the first step of DC vaccination strategies is to arm DCs with tumor-specific antigens. This issue has successfully been achieved by either culturing ex vivo DCs [16–17] from bone marrow precursors or more recently by targeting in vivo DC receptors with specific mAbs conjugated to tumor antigens [18–19]. In both cases, the development of a powerful DCs-based vaccination protocol requires a careful evaluation of the exact conditions necessary for their optimal maturation into potent immunostimulatory APCs. In particular, a strict control must be exercised over the form of the antigen loaded onto DCs, the antigen quantity, the persistence, the timing and the pathways essential for enhancing DC maturation and for licensing the antigen-loaded DCs in the T cell zone of lymph nodes [10].

Concerning the DCs maturation step, triggering C-type lectin receptors (CLRs) is crucial to enhance the antitumor immunity [10,20]. In particular, dendritic cell-specific ICAM-3 grabbing non-integrin (DC-SIGN), which belongs to the class of CLRs, is expressed mainly on the surface of immature DCs and plays a crucial role in the uptake of specific pathogens. DC-SIGN is able to bind in a Ca^2+^-dependent manner mannose and fucose residues on highly glycosylated proteins expressed on pathogens by means of its carbohydrate recognition domain (CRD) [21]. CLRs are antigen-uptake receptors. Moreover, the signaling pathways downstream induced by these receptors play a pivotal role in tailoring the immune response to break tumor-induced immunosuppression [22]. Therefore, a combination of DC-SIGN ligands and specific tumor-associated antigens could successfully target DCs and trigger an efficient antitumor response.

**Figure 1:**
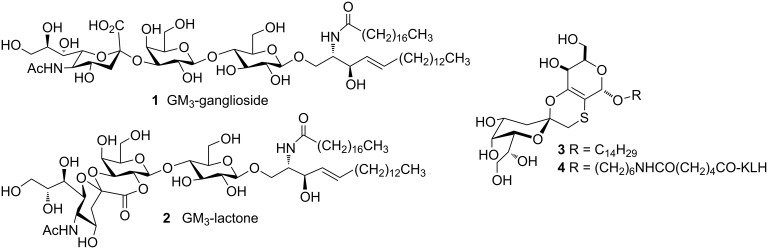
Structure of GM_3_-ganglioside **1**, GM_3_-lactone **2**, GM_3_-lactone mimetic **3**, and GM_3_-lactone mimetic conjugated to KLH protein (**4**).

Melanoma has long been considered a promising target for immunotherapeutic approaches and has been a major focus of clinical development efforts in the realm of immunotherapy [23–25]. GM_3_-ganglioside **1** (Figure 1), the major glycosphingolipid in normal melanocytes, is overexpressed in melanoma cells with metastatic potential [26–27]. It has been considered a carbohydrate melanoma-associated antigen and widely investigated as a key component of a potential vaccine against melanoma disease [28].

The GM_3_ metabolite, named GM_3_-lactone **2** (Figure 1) has also been found in melanoma cells as a minor component [29–30]. Although more immunogenic than GM_3_-ganglioside **1**, GM_3_-lactone **2** failed as an immunostimulant because under physiological conditions the available amount of lactone is below the recognition threshold and therefore scarcely effective as an immunostimulant.

Several years ago [31], we reported on the conformational analysis and the synthesis of thioether **3** (Figure 1), a hydrolytically stable mimetic of the GM_3_-lactone **2**. Structurally simpler than the native antigen, the mimetic **3** presents the folded shape characteristics of the GM_3_-lactone and in addition it is stable under physiological conditions [31]. We conjugated the mimetic **3** to the immunogenic protein KLH and demonstrated that the corresponding KLH-glycoconjugated **4** (Figure 1) was able to elicit in vivo antimelanoma antibodies [32].

More recently [33], we established that the multivalent presentation of this synthetic mimetic positively interferes with human melanoma cell (A375) adhesion, migration and resistance to apoptosis, showing a clear amplification of the biological properties of the monovalent synthetic antigen as an immunomodulator as well as an anti-adhesive agent in melanoma progression.

**Scheme 1:**
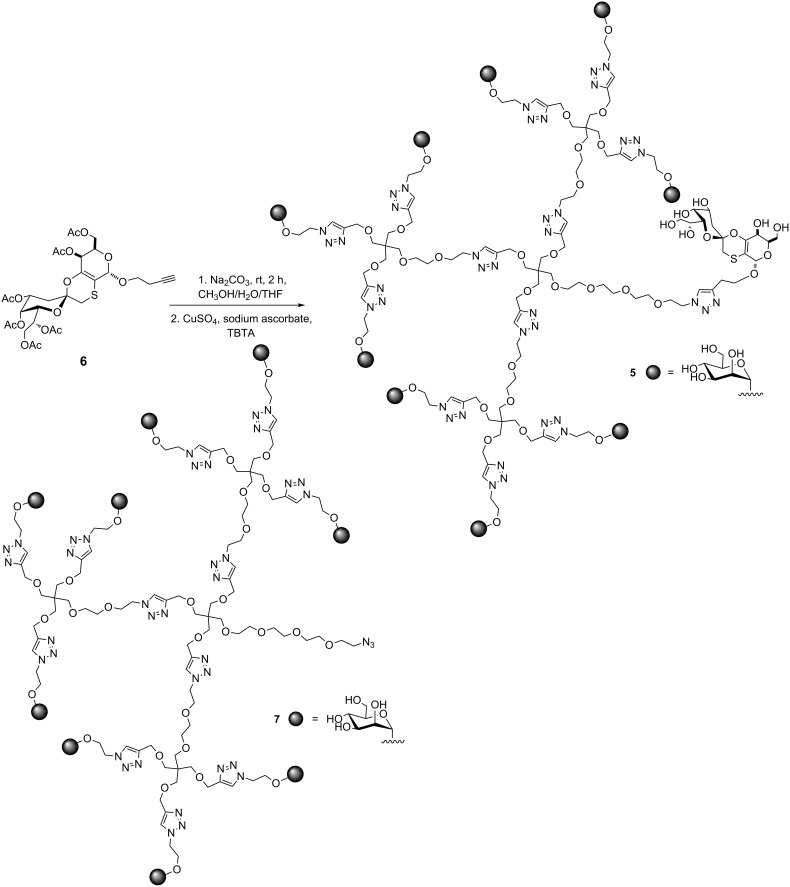
Synthesis of the bifunctional multivalent glycodendron **5**.

Taking into account all these data and relying on recent results on the use of mannose-based glycodendrons as vectors for antigen delivery to DCs [34], we report here on the convergent synthesis of the bifunctional multivalent glycodendron **5** (Scheme 1) and on human DC activation and related mixed-lymphocyte reaction (MLR) induced by the antigenic glycodendron **5**.

## Results and Discussion

Glycodendron **5** (Scheme 1) is a bifunctional compound containing nine residues of mannose for DC targeting and one residue of the mimetic **3** as a carbohydrate melanoma-associated antigen. We have previously demonstrated that a glycodendron bearing nine copies of the monosaccharide mannose can be taken up by DCs in a receptor-dependent manner by means of the lectin DC-SIGN [34]. This dendron has the adequate size and valency to efficiently interact with this receptor. Dendron **7** (Scheme 1), presenting an azido group at the focal position, was synthesized as previously described [35]. This functionalization permits, in a further step, the conjugation of any molecule conveniently functionalized with an alkyne group by a Cu(I) azide–alkyne cycloaddition (CuAAC) reaction. Then, the mimetic **6** with a butyne group at the anomeric position, which is required for the conjugation to the glycodendron **7** (Scheme 1), was also prepared as already reported [33]. The synthesis of the tricyclic spiro unit of **6** was efficiently performed relying on a totally diastereoselective inverse electron-demand [4 + 2] hetero-Diels–Alder reaction , as described in [31].

The synthesis of compound **5** is depicted in Scheme 1. The mimetic **6** was deprotected with sodium carbonate at room temperature. Without further purification the resulting syrup was conjugated with the glycodendron **7** by a CuAAC reaction with CuSO_4_ as a copper source, sodium ascorbate to reduce Cu(II) to Cu(I) in situ, and tris[(1-benzyl-1*H*-1,2,3-triazol-4-yl)methyl]amine (TBTA) to stabilize Cu(I). The solution was treated with a resin (Quadrasil MP) to remove any trace of copper that could cause interferences in the biological assays. After purification by size exclusion chromatography by using a LH-20 column, the bifunctional glycodendron **5** was obtained in 86% yield and characterized by NMR and MS (electrospray).

**Figure 2:**
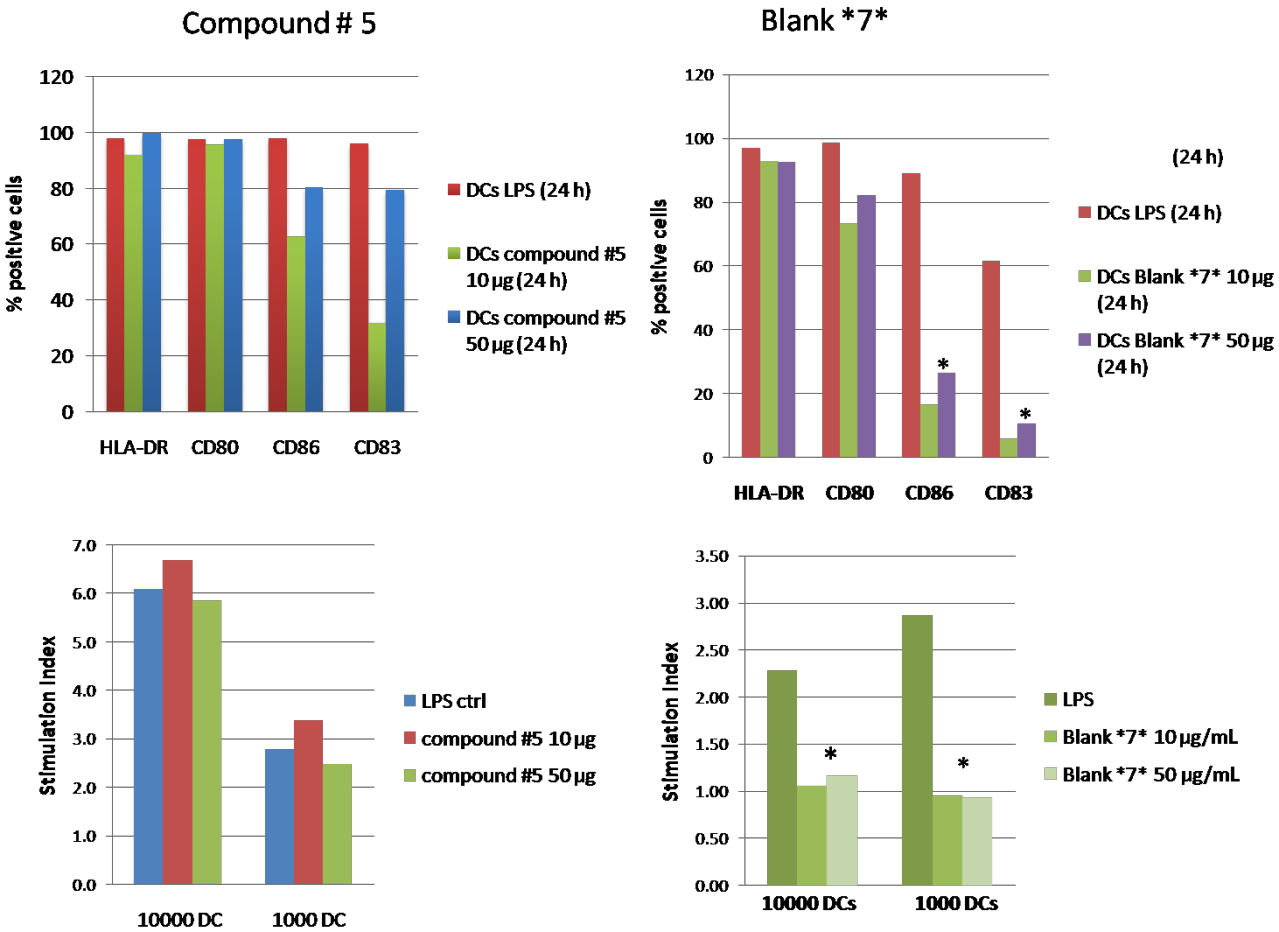
Upper panels: percentage of expression of dendritic cell markers (HLA-DR ECD, CD80 FITC, CD86 PE and CD83 PC5). Cell phenotypes, expressed as the percentage of positive cells for CD80, CD86 and HLA-DR, did not change when activation took place in the presence of compound **5** (left) at 10 or 50 µg/mL compared to LPS. CD83, an activation marker, was fully expressed at the higher dose of treatment. Compound **7** did not activate DCs when compared to LPS (right), differences between CD83 and CD86 expression are statistically significant at the two doses tested (*p < 0.05). One experiment is representative of three independent ones. Lower panels: mixed lymphocytes reaction (MLR). Compound **5** (left) at the dose of 10 or 50 µg/mL did not affect T lymphocyte proliferative response. As expected from the phenotype, scaffold (right) treated DCs at the dose of 10 or 50 µg/mL, significantly reduces the proliferative response (*p < 0.05). One experiment is representative of three independent ones. The analysis was performed by an unpaired t-test indicating that the mean is statistically significant at *p* < 0.05.

We tested in vitro human myeloid DCs (see methods) for an activation with LPS (positive control, 1 µg/mL), the bifunctional multivalent glycodendron **5** and **7** (negative control). Two doses (10 µg and 50 µg) of each compound were used. Our data showed that **5**, but not **7**, induces DC activation in terms of phenotype expression of MHC class II molecules, CD80 and CD86 co-stimulatory molecules and CD83 activation marker, as observed with positive control (Figure 2, upper panels, one experiment representative of three independent ones). To test the functional activity of the differently treated DCs, we performed on the same cells mixed lymphocytes reactions (MLR) (see Experimental) and checked the proliferative T lymphocyte response after allo-stimulation. As depicted in Figure 2, DCs treated with LPS or glycodendron **5** fully stimulate T lymphocytes, whereas **7** does not stimulate T lymphocytes (Figure 2, lower panel, one experiment representative of three independent experiments).

In the development of immunotherapy as an emerging strategy to treat tumours, DCs are an object of great interest because they can be used as APCs to stimulate the immune system against a specific tumor antigen. For this purpose, DCs can be pulsed ex vivo with the corresponding tumor antigen. However, this strategy is complicated and expensive, requiring the isolation of patients’ DCs, the pulsing of DCs with the antigen, and their reinsertion. Another approach envisages the targeting of DCs in vivo by using a selective vector combined with a cargo tumor antigen. This second strategy requires a system which should be able to selectively target DCs in vivo. In this work, we have shown how to combine in a single entity a mannosylated dendron able to selectively interact with DCs through DC-SIGN armed with a synthetic antigen. This ditopic glycodendron **5** demonstrated to be correctly designed to activate dendritic cells and stimulate T cells. Biological data clearly showed that the multivalent glycodendron **5** activates human immature DCs, induces the expression of all co-stimulatory molecules and MHCII, whereas the negative control **7** does not. Indeed, DCs need both MHC II and co-stimulation properly expressed on their surface to correctly integrate signals and activate T lymphocytes. If DCs do not sufficiently express one or more activation factors, their function may be impaired [36]. From a functional point of view, DCs activated by **5** stimulate T lymphocytes so that they proliferate in a classical MLR assay as LPS-activated cells (golden standard). When we tested the scaffold molecule alone (blank **7** in Figure 2) the phenotypic expression of DCs of co-stimulatory molecules and their T cell allo-stimulation ability was significantly impaired. Based on the data gathered in the experiments outlined here we conclude that the maturation/stimulation of DCs is specifically linked to the presence of the mimetic antigen residue and not determined by the scaffold alone.

## Conclusion

Here, we reported on the convergent synthesis of the ditopic multivalent glycodendron **5**, which contains an immunogenic mimetic of a carbohydrate melanoma associated antigen. We demonstrated that the immunogenic carbohydrate-based mimetic is able to induce human DCs activation if properly presented to DCs. Moreover, we showed that this activation is mediated by a permannosylated dendron interacting with the surface receptor DC-SIGN. These promising and preliminary biological results pave the way to the design of glycodendritic structures bearing antigen cargos as a selective vector to target APCs for stimulating immune responses. Further experiments must be performed to verify that the DCs activated by the multivalent ditopic glycodendron **5** are able to induce a strong pro-inflammatory response in vivo, thereby breaking the tolerance toward self antigens as melanoma and bypassing the tolerogenic environment normally established by the tumor activity. We envisage that this kind of compounds based on multivalent ditopic glycodendrons might be used to address the preparation of a synthetic vaccine against melanoma. In addition, this strategy might be applied to other diseases in immunotherapy.

## Experimental

Reagents were purchased from Sigma–Aldrich and Fluka and were used without purification. Synthetic compounds were purified by Sephadex (LH20). Thin-layer chromatography (TLC) was carried out with pre-coated Merck F_254_ silica gel plates. Reaction completion was observed by TLC with phosphomolibdic acid, 10% sulfuric acid in methanol or anisaldehyde as development reagents. ^1^H NMR and ^13^C NMR spectra were recorded on a Bruker Avance DRX 500 MHz spectrometer. Chemical shifts (δ*)* for ^1^H NMR and ^13^C NMR spectra are expressed in ppm relative to the residual solvent signal according to the indirect referencing method of the manufacturer. Signals are abbreviated as s, singlet; bs, broad singlet; d, doublet; t, triplet; q, quartet; m, multiplet. Mass spectra were obtained with a Bruker ion-trap Esquire 6000 apparatus (ESI).

### Synthesis

The preparation of compounds **6** [33] and **7** [35] was realized as previously described.

#### Synthesis of Glycodendron **5**

To a solution of mimetic **6** (0.006 g, 0.009 mmol) in a mixture of methanol/THF/water (1:1:1, 1.2 mL), sodium carbonate (46 mg, 0.40 mmol) was added. The solution was stirred at room temperature for 2 h. After that, resin IRA-120 H^+^ was added to reach pH 5. The reaction mixture was filtrated, and the solvent evaporated. The resulting syrup, glycodendron **7** (30 mg, 0.008 mmol), CuSO_4_·5H_2_O (0.4 mg, 0.001 mmol), TBTA (2.5 mg, 0.004 mmol) and sodium ascorbate (1 mg, 0.004 mmol) were dissolved in 0.8 mL of THF/H_2_O 1:1. After 2.5 h, a small amount of metal scavenger resin (Quadrasil MP) was added, and after further 5 minutes under stirring at room temperature the mixture was filtered on a cotton pad and the solution was purified by size exclusion chromatography (LH-20, MeOH 100%), furnishing 28.4 mg (86% yield, calculated over two steps) of the glycondendron **5** as a white foam. [α]^25^_D_ +23.9 (*c* 1, H_2_O/MeOH 1:1)

^1^H NMR (500 MHz, D_2_O) δ 8.01 (s, 9H, H_triazol_), 7.96 (s, 3H, H_triazol_), 7.89 (s. 1H, H_triazol_), 5.09 (s, 1H, H-1_gal_), 4.85 (s, 9H, H-1_mann_), 4.65–4.55 (m, 18H, O_mann_CH_2_CH_2_N), 4.56–4.45 (m, 32H, OCH_2_C_triazol_, O_linker_CH_2_C*H*_2_N, OC*H*_2_C_triazol_), 4.25–4.21 (m, 1H, H-3_sial.ac_), 4.08–4.04 (m, 9H, O_mann_C*H*_2_CH_2_N), 3.91–3.86 (m, 15H, O_mann_C*H*_2_CH_2_N, O_linker_C*H*_2_CH_2_N), 3.85–3.83 (m, 9H, H-2_mann_), 3.75–3.43 (m, 79H, H-6_mann_, H-3_mann_, H-5_mann_, O_pentaerythritol_C*H*_2_CH_2,_ O_pentaerythritol_CH_2_C*H*_2_, OCH_2linker_, C*H*_2_O_1gal_, H-4_gal_, H-5_gal_, H-6_gal_, H-4_sial.ac_, H-5_sial.ac_, H-6_sial.ac_, H-7_sial.ac_), 3.39–3.33 (m, 24H, CH_2pentaerythritol_), 3.30–3.27 (m, 8H, CH_2pentaerythritol_), 3.09–3.04 (m, 9H, H-4_mann_), 2.99–2.95 (m, 4H, SC*H*_2_, C_triazol_C*H*_2_CH_2_O), 2.04–1.97 (m, 1H, H-2_sial ac_), 1.91–1.84 (m, 1H, H-2_sial ac_); ^13^C NMR (125 MHz, D_2_O) δ 144.3 (C_triazol_), 142.4 (O-*C*=C-S), 125.3 (C_triazol_), 105.3 (O-C=*C*-S), 99.6 (C-1_mann_), 95.8 (C-1_gal_), 92.7 (C-1_sial.ac._), 72.8 (C-4_mann_), 70.4 (C-3_mann_), 69.91 (C-2_mann_), 69.7 (*C*H_2pentaerythritol_O_linker_), 68.9 (*C*H_2_O), 68.8 (*C*H_2_O), 68.7 (*C*H_2_O), 68.7 (*C*H_2_O), 68.4 (*C*H_2pentaerythritol_), 66.4 (C-5_mann_), 65.8 (*C*HO), 65.5 (O_mann_*C*H_2_CH_2_N, O*C*H_2_CH_2_N), 63.6 (O*C*H_2_C_triazol_), 60.7 (C-6_mann_), 50.0 (O_mann_CH_2_*C*H_2_N, OCH_2_*C*H_2_N, OCH_2_*C*H_2_N), 44.8 (C_pentaerythritol_), 35.4 (C-2_sial.ac._), 32.9 (S*C*H_2_), 35.3 (C_triazol_*C*H_2_CH_2_); ESIMS (*m*/*z*): calcd for C_166_H_269_N_39_O_86_S, 4217; found, 2142.1 [M + 2Cl)^2−^, 1440.4 [M + 3Cl]^3−^.

### Dendritic cell activation assay

#### Cells

DCs were generated from human monocytes of healthy donors as previously described [37]. Briefly, anti-CD14+ monocytes were positively sorted by magnetic microbeads (Miltenyi Biotec). Monocytes (at the density of 1 × 10^6^ cells/mL) were cultured for 6 days in medium (complete RPMI, 10% FCS) supplemented with GM-CSF (1000 U/mL, Labogen) and IL-4 (1000 U/mL, Labogen), At day 7, DCs were activated by 24 hours of incubation with LPS (1 µg/mL, Sigma–Aldrich), compound **5** and scaffold **7** (two doses, 50 and 10 µg/mL).

#### DC phenotype

The DC phenotype was analyzed by flow cytometry with a 4 color EpicsXL cytometer (Beckman–Coulter), equipped with Expo 32 software. Cell surface markers were labelled with monoclonal antibodies (Immunotech) directed against the following antigens (the tags are given in parentheses): CD80 (FITC), CD86 (PE), HLA-DR (ECD), CD83 (PC5). Cell vitality was tested with propidium iodide (PI, molecular probes). The cells were labelled in PBS with 1% FCS for 15 min at room temperature (rt), washed twice and immediately analyzed. For each test at least 10000 events were acquired.

#### Mixed lymphocyte reaction (MLR)

CD4+ T cells were negatively selected from peripheral blood mononuclear cells (PBMCs) by using the T cell isolation kit II from Miltenyi Biotec. Mixed lymphocyte reaction (MLR) was performed in 96-well U bottom plates (Nunc). 1 × 10^5^ CD4+ T cells were incubated for 5 days in RPMI with 10% FCS together with 1 × 10^4^ to 1 × 10^3^ allogeneic DCs. Experiments were conducted in quadruplicate. At day 5, the proliferative response was measured by the [3H]-thymidine ([3H]-Thy, 1 µCi/mL, Amersham) incorporation test. [3H]-Thy was added for the last 8 h of incubation. Plates were then harvested (TomtecMacIII) on glass fiber filters (Perkin Elmer), and [3H]-Thy uptake was measured by liquid scintillation in a Microbeta 1450 Trimux counter (Wallac). The proliferative response is reported as a stimulation index (SI, mean cpm response/mean cpm background).

#### Statistics

Data were expressed as mean + SD values. Statistical analysis was performed by using Student’s t-test where appropriate.
